# Wavelet analysis of postural stability reveals age-related differences in spectral response patterns during adaptation to immersive virtual reality

**DOI:** 10.1038/s41598-026-57978-1

**Published:** 2026-06-22

**Authors:** Ulrik Röijezon, Eva Ekvall Hansson, Jenny Älmqvist Nae, Elin Östlind, Jimmy Falk, Mitesh Patel, Rolf Johansson, Per-Anders Fransson

**Affiliations:** 1 Department of Health Sciences, Lund University, Lund, Sweden; 2 Department of Health, Education and Technology, Luleå University of Technology, Luleå, Sweden; 3 Department of Otorhinolaryngology Head and Neck Surgery, Skåne University Hospital, Lund, Sweden; 4 Bangor University, North Wales Medical School, Bangor, UK; 5 Department of Automatic Control, Lund University, Lund, Sweden; 6 Department of Clinical Sciences Lund, Lund University, Lund, Sweden

**Keywords:** Ageing, Postural stability, Spectral domain, Virtual Reality, Vision, Health care, Neuroscience

## Abstract

Current research indicates that Virtual Reality (VR) can serve as an effective tool for evaluating and training postural control responses and its processing, to distorted sensory input. The aim was to evaluate posturographic spectral responses and adaptation to repeated visual VR-stimulation in young and older adults with wavelet analysis. Twenty-eight young (mean 25.3 years) and 25 older (mean 74.8 years) adults were included. Participants were standing on a force plate performing two control tests (eyes open and closed) and thereafter repeatedly watched a 120-second VR-simulation of a roller-coast ride five times. The first VR session produced a marked two-fold stability response: (1) significant spectral energy increased within 0.4–8.5 Hz in anteroposterior and lateral directions, and (2) significant spectral energy decreased within 0.03–0.13 Hz in anteroposterior direction. Older adults used significantly more high frequency energy and less low frequency energy. Repeated VR sessions significantly decreased high frequency energy in both groups. Wavelet analysis indicates that both younger and older adults employed similar spectral response patterns in response to immersive visual stimulation. However, older adults showed larger shifts in spectral characteristics, suggesting age-related differences in resilience. Postural control appeared capable of rapidly adapting to adjust biomechanical strategies and sensory weighting.

## Introduction

To maintain stability in challenging conditions, individuals across all ages engage adaptive mechanisms to strengthen the resilience of postural control and its ability to modify performance characteristics^1–3^. Such robustness is achieved through the continuous and independent acquisition of multi-sensory inputs from different sensory systems. Each system, with its unique receptive properties, monitors the stability and movement of the biomechanically complex, multi-segmented human body^4^. A control system that integrates multisensory input gains further resilience by selecting and reweighting sensory sources to extract the most accurate information^2,5^.

To maintain postural control, the central nervous system (CNS) primarily applies two movement control methods: feedback and feedforward control. When using a feedback control approach, the system gathers sensory information before generating a response. A well-functioning feedback system, with a rapid information-to-response processing loop, can almost always reliably produce appropriate reactions during simple or moderately complex tasks - such as walking across uneven terrain - without compromising stability. The feedback control principles are further enhanced for human postural control in that different sensory systems provide feedback information within different spectral bandwidths, i.e., of very fast movements by proprioception and mechanoreceptive sensors and of static position, and slow movements by the vestibular system and vision^6^. These properties produce non-linear system control characteristics within the spectral domain, where low frequency information from vision reduces the postural sway activity mainly above the spectral bandwidth of visual information^2,7^. In contrast, feedforward control involves the generation of anticipatory actions based on predictive information. When sufficient cues are available, individuals can prepare to optimize performance in advance of upcoming tasks, such as adjusting gait and body posture when approaching stairs^2,4^. When performing challenging new or unpredictable tasks, a common strategy is to increase muscle co-contraction and thereby joint impedance^8^.

Vision is a vital source of information for both feedback and feedforward control and thus plays an essential role in maintaining postural stability^9,10^. However, several age-related and medical conditions can affect the quality of visual information received, such as impaired vision^11^, acute vestibular loss^12^ and central disorders^13,14^. The accuracy and resolution of other multisensory information can also be reduced from medical disorders and by degenerative aging processes, e.g., from increased receptor stimulation thresholds, which delay the detection of movement^15^. When key sensory inputs are lost or disrupted, it is essential that the CNS can identify the source of sensory mismatches and extract the most accurate information from alternative sensory channels^4^. Recent research has suggested that rehabilitation involving repeated exposure to Virtual Reality (VR) may help address sensory mismatch issues, particularly those in which visual input plays a dominant role^16–19^. Effective sensory reweighting and adaptation is demonstrated as postural stability is maintained despite visual disturbances^18,20^. Adaptation is a crucial component of postural control, as it allows sensorimotor programs to be refined and stabilized by incorporating information gained from previous, similar experiences^21,22^.

Postural stability is commonly evaluated experimentally using posturography, often in combination with experimentally induced balance perturbations. These may include sensory manipulations such as vibratory proprioceptive stimulation, galvanic vestibular stimulation, or exposure to altered visual environments, as well as mechanically induced perturbations such as translations of the support-surface movements or surface tilts^18,23,24^. Previous research has shown that posturography can effectively assess and quantify the level of challenge experienced by both healthy individuals and patients when subjected to challenging balance conditions involving altered sensory input^25,26^. Traditionally, posturography is analyzed by quantifying e.g., the magnitude or the speed of the COP excursions, or using dichotomized frequency analyses. However, some prior studies have suggested that wavelet analyzes can potentially be a more sensitive method when analyzing the frequency characteristics of the CoP-oscillations^1,27,28^.

To our knowledge, this study is the first study to use posturography in combination with wavelet analysis to investigate whether exposure to VR elicits comparable responses among healthy young and older adults, and whether it induces similar adaptive changes in the spectral characteristics of postural control. This approach can increase our understanding of postural stability by increasing the resolution of the posturographic spectral analyses, compared to commonly used dichotomized methods^18,20,29^.

The main aim of this study was to investigate how the spectral characteristics of postural control in healthy young and older adults respond to exposure to challenging visual environments by immersive Virtual Reality, and whether such repeated exposures induce adaptive changes in the spectral profile of postural control. The hypotheses were that younger and older adults would exhibit different spectral responses to VR stimulation, and that repeated exposure across separate sessions would lead to adaptive changes that gradually help participants reduce the impact of visually induced postural alterations.

## Results

### Effects of repetition and age group on postural stability during VR sessions

**Fig. 1:**
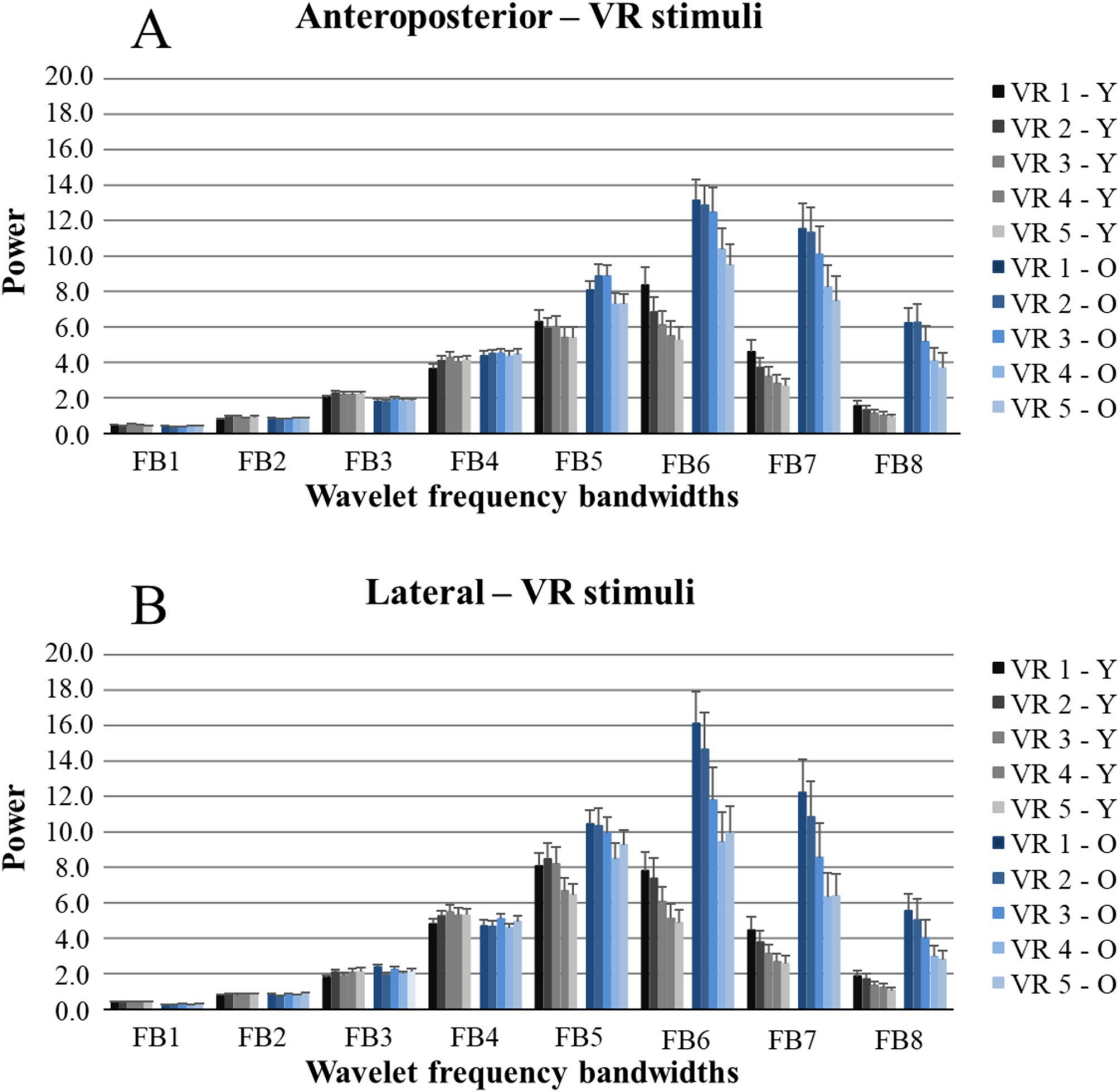
**A)** Anteroposterior stability in young and older adult groups during repeated VR sessions as reflected by wavelet analysis. The performance recorded during the five repeated VR sessions is presented as bars (mean) and whiskers (SEM) values for 8 frequency bandwidths (FB), see bandwidth details in Table 1. Results for the young group are denoted with Y and results for the older group are denoted with O. Figure 1B**)** Lateral stability in the young and older adult groups during repeated VR sessions as reflected by wavelet analysis.

When the participants repeatedly watched the same VR movie, the wavelet values gradually decreased significantly across sessions in the high frequency bandwidths (FB) FB5 – FB8 in anteroposterior (*p* ≤ 0.007) direction (Fig. 1A; Table 1). In lateral direction, the wavelet values also gradually decreased significantly across sessions in the FB5 – FB8 bandwidths (*p* < 0.001) but also increased in the low FB1 bandwidth (*p* = 0.007) (Fig. 1B; Table 1).

In anteroposterior direction, the wavelet values were significantly higher in the older group than in the younger group in the FB5 – FB8 bandwidths (*p* ≤ 0.006), but significantly lower in the FB1 and FB3 bandwidths (*p* ≤ 0.032). In lateral direction, the wavelet values were significantly higher in the older group than in the younger group in the FB5 – FB8 bandwidths (*p* ≤ 0.040), but again significantly lower in the FB1 bandwidths (*p* < 0.001).

The significant interactions between repetition and age group revealed that the older group significantly decreased the wavelet values more than the young group in the FB8 bandwidth (*p* = 0.035) and increased the wavelet values more in the FB2 bandwidth (*p* = 0.013) in anteroposterior directions (Table 1). In lateral direction, the older group significantly increased the wavelet more in the FB1 – FB3 bandwidths (*p* ≤ 0.040) than the younger group.

**Table 1:** Effects of repetition and age group on postural stability during VR sessions.

Wavelet ^a^	Anteroposterior ^b, c^			Lateral ^b, c^		
Frequency ranges (Hz)	Repetition	Age group	Repetition x Age group	Repetition	Age group	Repetition x Age group
FB1 (0.028–0.067)	0.459 [0.6]	**0.032 [4.9]**	0.063 [3.6]	**0.007 [8.0]**	**< 0.001 [24.9]**	**0.040 [4.4]**
FB2 (0.055–0.133)	0.140 [2.3]	0.139 [2.3]	**0.013 [6.7]**	0.346 [0.9]	0.887 [0.0]	**0.014 [6.4]**
FB3 (0.110–0.266)	0.317 [1.0]	**0.016 [6.2]**	0.126 [2.4]	0.498 [0.5]	0.413 [0.7]	**0.016 [6.3]**
FB4 (0.219–0.531)	0.196 [1.7]	0.209 [1.6]	0.205 [1.6]	0.273 [1.2]	0.315 [1.0]	0.405 [0.7]
FB5 (0.438–1.060)	**0.007 [7.9]**	**0.006 [8.4]**	0.185 [1.8]	**< 0.001 [19.2]**	**0.040 [4.4]**	0.440 [0.6]
FB6 (0.88–2.12)	**< 0.001 [42.9]**	**< 0.001 [14.7]**	0.058 [3.8]	**< 0.001 [55.2]**	**0.003 [10.0]**	0.300 [1.1]
FB7 (1.75–4.25)	**< 0.001 [58.1]**	**< 0.001 [25.0]**	0.114 [2.6]	**< 0.001 [71.8]**	**< 0.001 [15.2]**	0.150 [2.1]
FB8 (3.51–8.50)	**< 0.001 [64.6]**	**< 0.001 [33.9]**	**0.035 [4.7]**	**< 0.001 [64.1]**	**< 0.001 [17.7]**	0.166 [2.0]

^a^ Repeated measures GLM ANOVA analyses of how the postural stability during VR was affected by main factor “Repetition”, “Age group” and the interaction factor “Repetition” x “Age group”.

^b^ Blue color represents that the main factor or factor interactions significantly decreased the wavelet values, and red color represents that the main factor or factor interactions significantly increased the wavelet values.

^c^ Presented as: p-values and [F-values]. The notation “< 0.001” means that the p-value is smaller than 0.001

## Initial effects and adaptation to VR in the young group

**Fig. 2:**
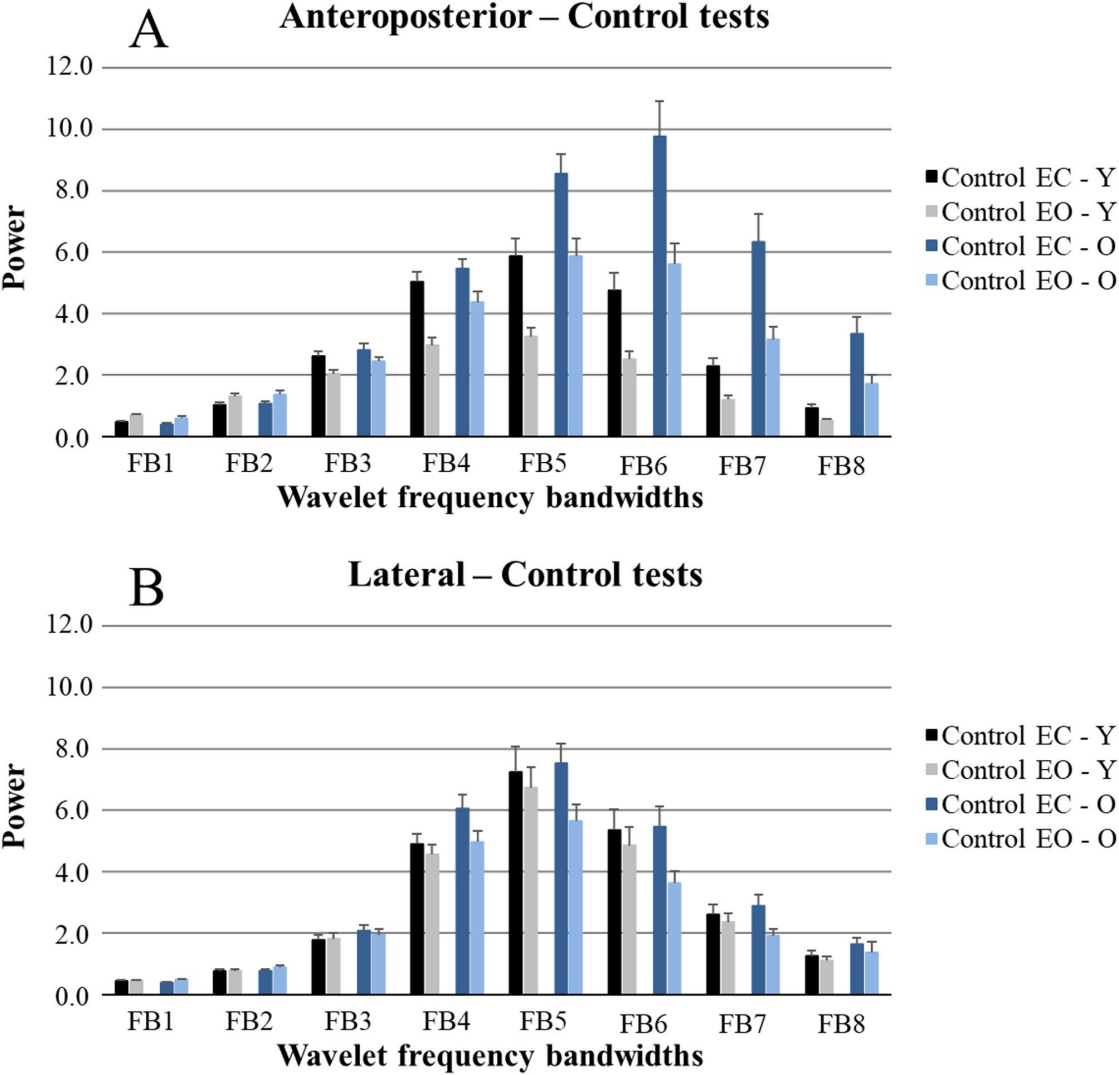
**A)** Anteroposterior stability in the young and older adult groups during the control tests as reflected by wavelet analysis. Figure 2B**)** Lateral stability in the young and older adult groups during the control tests as reflected by wavelet analysis.

The young group had significantly higher wavelet values during VR session 1 vs. the control test with eyes open, in FB5 – FB8 bandwidths (mean 201% higher, *p* < 0.001) but significantly lower values in the FB1 – FB2 bandwidths (38% lower, *p* < 0.001) in anteroposterior direction (Fig. 1A repeated VR, Fig. 2A control tests, Table 2). In lateral direction, the wavelet values were significantly higher in the FB5 – FB8 bandwidths (60% higher, *p* ≤ 0.025) (Fig. 1B repeated VR, Fig. 2B control tests, Table 2).

**Table 2:** Initial effects and adaptation to VR in the young group.

Wavelet changes ^a^		*p*-value	VR 1/EO ^b^	*p*-value	VR 5/VR 1 ^c^	*p*-value	VR 5/EO ^d^
Anteroposterior ^e^	FB1 (0.028–0.067)	**< 0.001**	**0.66 (3.80)**	0.362	0.93 (0.15)	**< 0.001**	**0.62 (0.68)**
	FB2 (0.055–0.133)	**< 0.001**	**0.58 (2.69)**	**0.030**	**1.20 (0.07)**	**0.002**	**0.70 (0.67)**
	FB3 (0.110–0.266)	0.902	0.98 (3.40)	0.202	1.11 (0.06)	0.171	1.09 (1.48)
	FB4 (0.219–0.531)	0.077	1.23 (3.80)	**0.043**	**1.13 (0.15)**	**< 0.001**	**1.39 (0.68)**
	FB5 (0.438–1.060)	**< 0.001**	**1.94 (2.69)**	0.227	0.86 (0.07)	**< 0.001**	**1.67 (0.67)**
	FB6 (0.88–2.12)	**< 0.001**	**3.33 (3.40)**	**< 0.001**	**0.63 (0.06)**	**< 0.001**	**2.10 (1.48)**
	FB7 (1.75–4.25)	**< 0.001**	**3.84 (3.80)**	**< 0.001**	**0.58 (0.15)**	**< 0.001**	**2.23 (0.68)**
	FB8 (3.51–8.50)	**< 0.001**	**2.94 (2.69)**	**< 0.001**	**0.59 (0.07)**	**< 0.001**	**1.75 (0.67)**
Lateral ^e^	FB1 (0.028–0.067)	0.295	0.87 (0.10)	0.522	1.08 (0.15)	0.646	0.94 (0.08)
	FB2 (0.055–0.133)	0.678	1.00 (0.10)	0.362	1.08 (0.12)	0.150	1.08 (0.08)
	FB3 (0.110–0.266)	0.884	0.99 (0.12)	0.157	1.18 (0.11)	0.264	1.17 (0.15)
	FB4 (0.219–0.531)	0.386	1.06 (0.12)	0.327	1.10 (0.12)	0.218	1.17 (0.16)
	FB5 (0.438–1.060)	**0.025**	**1.20 (0.13)**	**0.017**	**0.80 (0.09)**	0.711	0.96 (0.11)
	FB6 (0.88–2.12)	**< 0.001**	**1.61 (0.22)**	**< 0.001**	**0.63 (0.08)**	0.438	1.01 (0.15)
	FB7 (1.75–4.25)	**< 0.001**	**1.89 (0.31)**	**< 0.001**	**0.58 (0.08)**	0.695	1.09 (0.22)
	FB8 (3.51–8.50)	**0.001**	**1.69 (0.31)**	**< 0.001**	**0.58 (0.07)**	0.218	0.98 (0.16)

^a^ Presented as quotient between the mean values for each dataset (SEM of individual quotients on sample level).

^b^ Quotient between VR session 1/control test with eyes open.

^c^ Quotient between VR session 5/VR session 1.

^d^ Quotient between VR session 5/control test with eyes open.

^e^ Blue color represents that VR decreased the wavelet values, and red color represents that VR increased the wavelet values.

The young group significantly reduced the wavelet values across the five VR sessions in the FB6 – FB8 (40% decrease, *p* < 0.001) but significantly increased the wavelet values in the FB2 and FB4 bandwidths (17% increase, *p* ≤ 0.043) in anteroposterior direction (Table 2). In lateral direction, the wavelet values were significantly reduced in the FB5 – FB8 bandwidths (35% lower, *p* ≤ 0.017) (Table 2).

When comparing the wavelet values after adaptation in VR session 5 with the wavelet values during the control test with eyes open, the young group had significantly higher wavelet values in the FB4 – FB8 bandwidths (83% higher, *p* < 0.001) but significantly lower values in the FB1 – FB2 bandwidths (34% lower, *p* ≤ 0.002) in anteroposterior direction (Table 2).

## Initial effects and adaptation to VR in the older group

The older group had significantly higher wavelet values during VR session 1 vs. the control test with eyes open, in the FB5 – FB8 bandwidths (mean 176% higher, *p* < 0.001) but significantly lower values in the FB1 – FB3 bandwidths (34% lower, *p* ≤ 0.015) in anteroposterior direction (Fig. 1A repeated VR, Fig. 2A control tests, Table 3). In lateral direction, the wavelet values were significantly higher in the FB3 and FB5 – FB8 bandwidths (258% higher, *p* ≤ 0.034) but significantly lower in the FB1 bandwidth (55% lower, *p* < 0.001) (Fig. 1B repeated VR, Fig. 2B control tests, Table 3).

**Table 3:** Initial effects and adaptation to VR in the older group.

Stability changes ^a^		*p*-value	VR 1/EO ^b^	*p*-value	VR 5/VR 1 ^c^	*p*-value	VR 5/EO ^d^
Anteroposterior ^e^	FB1 (0.028–0.067)	**< 0.001**	**0.66 (0.07)**	0.922	1.03 (0.15)	**0.046**	**0.68 (0.11)**
	FB2 (0.055–0.133)	**< 0.001**	**0.58 (0.08)**	0.663	1.05 (0.11)	**< 0.001**	**0.61 (0.07)**
	FB3 (0.110–0.266)	**0.015**	**0.74 (0.11)**	0.684	1.03 (0.10)	**0.004**	**0.76 (0.06)**
	FB4 (0.219–0.531)	1.000	1.00 (0.12)	0.922	1.02 (0.09)	0.726	1.02 (0.10)
	FB5 (0.438–1.060)	**< 0.001**	**1.38 (0.17)**	**0.039**	**0.91 (0.06)**	0.069	1.25 (0.21)
	FB6 (0.88–2.12)	**< 0.001**	**2.34 (0.29)**	**< 0.001**	**0.72 (0.07)**	**< 0.001**	**1.69 (0.38)**
	FB7 (1.75–4.25)	**< 0.001**	**3.65 (0.42)**	**0.001**	**0.65 (0.09)**	**< 0.001**	**2.36 (0.52)**
	FB8 (3.51–8.50)	**< 0.001**	**3.65 (0.50)**	**0.001**	**0.59 (0.09)**	**0.006**	**2.16 (0.55)**
Lateral ^e^	FB1 (0.028–0.067)	**< 0.001**	**0.45 (0.07)**	**0.002**	**1.41 (0.26)**	**0.001**	**0.63 (0.10)**
	FB2 (0.055–0.133)	0.958	0.96 (0.10)	0.331	1.03 (0.07)	0.790	0.99 (0.09)
	FB3 (0.110–0.266)	**0.034**	**1.22 (0.21)**	0.303	0.89 (0.08)	0.178	1.09 (0.14)
	FB4 (0.219–0.531)	0.833	0.95 (0.10)	0.360	1.05 (0.07)	0.726	1.00 (0.10)
	FB5 (0.438–1.060)	**< 0.001**	**1.85 (0.21)**	0.101	0.89 (0.07)	**< 0.001**	**1.64 (0.18)**
	FB6 (0.88–2.12)	**< 0.001**	**4.43 (0.82)**	**< 0.001**	**0.62 (0.07)**	**< 0.001**	**2.73 (0.42)**
	FB7 (1.75–4.25)	**< 0.001**	**6.33 (1.67)**	**< 0.001**	**0.52 (0.05)**	**< 0.001**	**3.31 (0.84)**
	FB8 (3.51–8.50)	**< 0.001**	**4.05 (1.34)**	**< 0.001**	**0.51 (0.08)**	**0.004**	**2.05 (0.76)**

^a^ Presented as quotient between the mean values for each dataset (SEM of individual quotients on sample level).

^b^ Quotient between VR session 1/control test with eyes open.

^c^ Quotient between VR session 5/VR session 1.

^d^ Quotient between VR session 5/control test with eyes open.

^e^ Blue color represents that VR decreased the wavelet values, and red color represents that VR increased the wavelet values.

The older group significantly reduced the wavelet values across the five VR sessions in the FB5 – FB8 bandwidths (28% lower, *p* ≤ 0.039) in anteroposterior direction (Table 3). In lateral direction, the wavelet values were significantly lower in the FB6 – FB8 bandwidths (45% lower, *p* < 0.001) but significantly higher in the FB1 bandwidth (41% higher, *p* = 0.002) (Table 3).

When comparing the wavelet values after adaptation in VR session 5 with the wavelet values during the control test with eyes open, the older group had significantly higher wavelet values in the FB6 – FB8 bandwidths (107% higher, *p* ≤ 0.006) but significantly lower values in the FB1 – FB3 bandwidths (32% lower, *p* ≤ 0.046) in anteroposterior direction (Table 3). In lateral direction, the wavelet values were significantly higher in the FB5 – FB8 bandwidths (143% higher, *p* ≤ 0.004) but significantly lower in the FB1 bandwidth (37%, *p* = 0.001) (Table 3).

## Effects of vision and age group on postural stability during control tests

Vision (i.e., eyes open) decreased the wavelet values significantly in the FB3 – FB8 bandwidths in anteroposterior (*p* ≤ 0.006) direction but increased the wavelets within the FB1 – FB2 bandwidths (*p* ≤ 0.002) (Fig. 2A; Table 4). In lateral direction, vision decreased the wavelet values significantly in the FB4 and FB6 – FB8 bandwidths (*p* ≤ 0.046) but increased in the FB1 bandwidth (*p* = 0.027) (Fig. 2B; Table 4).

**Table 4:** Effects of vision and age group on postural stability during control tests.

Wavelet ^a^	Anteroposterior ^b, c^			Lateral ^b, c^			
	Vision	Age group	Vision x Age group	Vision	Age group		Vision x Age group
FB1 (0.028–0.067)	**< 0.001 [29.3]**	**0.031 [5.0]**	0.824 [0.1]	**0.027 [5.2]**		0.779 [0.1]	0.408 [0.7]
FB2 (0.055–0.133)	**0.002 [10.7]**	0.781 [0.1]	0.948 [0.0]	0.130 [2.4]		0.551 [0.4]	0.491 [0.5]
FB3 (0.110–0.266)	**0.006 [8.1]**	0.113 [2.6]	0.360 [0.9]	0.494 [0.5]		0.340 [0.9]	0.813 [0.1]
FB4 (0.219–0.531)	**< 0.001 [51.1]**	**0.017 [6.1]**	**0.015 [6.4]**	**0.046 [4.2]**		0.096 [2.9]	0.502 [0.5]
FB5 (0.438–1.060)	**< 0.001 [79.8]**	**< 0.001 [15.7]**	0.196 [1.7]	0.050 [4.0]		0.880 [0.0]	0.154 [2.1]
FB6 (0.88–2.12)	**< 0.001 [85.8]**	**< 0.001 [23.1]**	0.584 [0.3}]	**0.010 [7.2]**		0.565 [0.3]	0.085 [3.1]
FB7 (1.75–4.25)	**< 0.001 [88.0]**	**< 0.001 [28.0]**	0.979 [0.0]	**0.005 [8.6]**		0.884 [0.0]	0.082 [3.1]
FB8 (3.51–8.50)	**< 0.001 [64.6]**	**< 0.001 [31.1]**	0.460 [0.6]	**0.020 [5.8]**		0.448 [0.6]	0.102 [2.8]

^a^ Repeated measures GLM ANOVA analyses of how the stability during VR was affected by main factor “Vision”, “Age group” and the interaction factor “Vision” x “Age group”.

^b^ Blue color represents that the main factor or factor interactions decreased the wavelet values, and red color represents that the main factor or factor interactions increased the wavelet values.

^c^ Presented as: p-values and [F-values].

In anteroposterior direction, the wavelet values were significantly higher in the older group than in the young group in the FB4 – FB8 bandwidths (*p* ≤ 0.017), but significantly lower in the FB1 bandwidth (*p* = 0.031) (Table 4).

The significant interactions between vision and age group revealed that the older group significantly increased the wavelet values more than the young group in the FB4 bandwidth (*p* = 0.015) in anteroposterior directions (Table 4).

## Effects of vision during the control tests in the young group

In the young group, the wavelet values were significantly higher with eyes closed than with eyes open in the FB3 – FB8 bandwidths (73% higher, *p* ≤ 0.004) but significantly lower in the FB1 – FB2 bandwidths (27% lower, *p* ≤ 0.026) in anteroposterior direction (Fig. 2A; Table 5).

**Table 5:** Effects of Vision during the control tests in the young group.

Wavelet changes ^a^	Anteroposterior ^c^		Lateral ^c^	
	*p*-value	EC/EO ^b^	*p*-value	EC/EO ^b^
FB1 (0.028–0.067)	**0.002**	**0.68 (0.08)**	0.831	0.97 (0.11)
FB2 (0.055–0.133)	**0.026**	**0.78 (0.10)**	0.831	1.00 (0.09)
FB3 (0.110–0.266)	**0.004**	**1.28 (0.11)**	0.797	0.98 (0.10)
FB4 (0.219–0.531)	**< 0.001**	**1.70 (0.16)**	0.508	1.07 (0.14)
FB5 (0.438–1.060)	**< 0.001**	**1.81 (0.19)**	0.522	1.08 (0.17)
FB6 (0.88–2.12)	**< 0.001**	**1.90 (0.21)**	0.451	1.10 (0.14)
FB7 (1.75–4.25)	**< 0.001**	**1.91 (0.21)**	0.438	1.11 (0.12)
FB8 (3.51–8.50)	**< 0.001**	**1.76 (0.22)**	0.598	1.13 (0.13)

^a^ Presented as quotient between the mean values for each dataset (SEM of individual quotients on sample level).

^b^ Quotient between the control tests with eyes closed /control tests with eyes open.

^c^ Blue color represents that Vision increased the wavelet values, and red color represents that Vison decreased the wavelet values.

### Effects of vision during the control tests in the older group

In the older group, the wavelet values were significantly higher with eyes closed than with eyes open in the FB4 – FB8 bandwidths (69% higher, *p* ≤ 0.005) but significantly lower in the FB1 – FB2 bandwidths (28% lower, *p* ≤ 0.015) in anteroposterior direction (Fig. 2B; Table 6). In lateral direction, the wavelet values were significantly higher with eyes closed than with eyes open in the FB4 – FB8 bandwidths (35% higher, *p* ≤ 0.037) but significantly lower in the FB1 bandwidth (18% lower, *p* = 0.039) (Fig. 2B; Table 6).

**Table 6:** Effects of Vision during the control tests in the older group.

Wavelet changes ^a^	Anteroposterior ^c^		Lateral ^c^	
	*p*-value	EC/EO ^b^	*p*-value	EC/EO ^b^
FB1 (0.028–0.067)	**< 0.001**	**0.67 (0.07)**	**0.039**	**0.82 (0.10)**
FB2 (0.055–0.133)	**0.015**	**0.78 (0.12)**	0.263	0.88 (0.08)
FB3 (0.110–0.266)	0.156	1.15 (0.23)	0.252	1.07 (0.10)
FB4 (0.219–0.531)	**0.005**	**1.25 (0.16)**	**0.037**	**1.22 (0.13)**
FB5 (0.438–1.060)	**< 0.001**	**1.46 (0.10)**	**0.016**	**1.33 (0.18)**
FB6 (0.88–2.12)	**< 0.001**	**1.74 (0.14)**	**0.007**	**1.51 (0.26)**
FB7 (1.75–4.25)	**< 0.001**	**2.01 (0.20)**	**0.015**	**1.50 (0.28)**
FB8 (3.51–8.50)	**< 0.001**	**1.97 (0.22)**	**0.015**	**1.20 (0.28)**

^a^ Presented as quotient between the mean values for each dataset (SEM of individual quotients on sample level).

^b^ Quotient between the control tests with eyes closed /control tests with eyes open.

^c^ Blue color represents that Vision increased the wavelet values, and red color represents that Vison decreased the wavelet values.

## Discussion

The main aim of the study was to use a wavelet method to investigate the spectral characteristics of postural control during VR-stimulation in healthy young and older adults, and investigate adaptive changes in the spectral profile over 5 repeated VR trials. In both groups of participants, the VR sessions produced a marked two-fold stability response: (1) the spectral energy increased significantly within the higher frequency bandwidths in the anteroposterior and lateral directions, and (2) the spectral energy decreased significantly within the lower bandwidths in anteroposterior direction. These changes were more pronounced in older adults, shown by more high frequency energy use and less low frequency energy use. Repeated sessions of VR significantly decreased spectral energy used within the high frequency bandwidths in both groups.

One major contribution of this study is detailing the kind of information that is gained from wavelet analyses of postural control during VR-stimulation. Interestingly, the wavelet analysis method revealed that most spectral responses did not occur within the low-frequency range typically associated with the receptive bandwidth of visual input^30^. This suggests that postural control does not operate solely according to a linear input-output model in the spectral domain. Another contribution of this study is that wavelet analyses detailed how postural control of young and older adults differ when exposed to VR-stimulation. The increased challenge of maintaining postural stability that was experienced by older adults was predominantly shown as elevated spectral energy in the 0.4–8.5 Hz range, and only partially by shifts in the 0.03–0.13 Hz range, which aligns with using a co-contraction strategy aimed at enhancing joint stiffness^8,30^. Hence, the wavelet analysis approach enabled us to (i) identify how effects were distributed across specific frequency ranges, (ii) verify that the observed differences were not driven by a single narrow band, and (iii) avoid potential bias introduced by arbitrary band selection. In our case, while the effects were ultimately robust at the level of broader frequency groupings, the higher-resolution analysis provided confidence in the consistency and structure of these effects across scales and repeated VR sessions.

It is important to note that a wavelet analysis does not mirror a Fast Fourier Transform (FFT) analysis. A key distinction is that wavelet decomposition divides spectral energy into non-linearly increasing frequency bands, where the lower frequencies are resolved in relatively narrow bands, while higher frequencies are represented by progressively broader bands. In addition, wavelet analysis quantifies signal energy across frequency ranges (e.g., 0.88–4.25 Hz) rather than within narrow, fixed-width frequency bands as in Fast Fourier analysis. Hence, wavelet scales represent band-limited but overlapping frequency responses, and therefore the associated power reflects aggregated signal content across a broader spectral region. Consequently, while traditional Fourier-based analyses of postural sway typically show that the majority of CoP power is concentrated below approximately 1 Hz, wavelet-derived estimates may distribute signal energy across adjacent higher-frequency bands due to the bandwidth limitations of each scale. This implies that apparent differences in the location of peak power across methods should not be interpreted as a shift in dominant physiological oscillations, but rather as a consequence of differences in spectral decomposition approaches. Furthermore, it should be noted that spectral measures of CoP reflect behavioral output and do not allow direct inference about underlying neuromuscular or sensory weighting mechanisms without complementary sensory (e.g., vestibular, proprioceptive, tactile) or motor (e.g., electromyography) tests.

The wavelet analysis method showed that the first exposure to the VR scene (session 1) elicited a comparable and pronounced dual-component response in the spectral domain in both young and older adults. In general, spectral energy significantly increased especially within the 0.4–8.5 Hz frequency range (FB5-FB8), while a significant decrease was observed within the 0.03–0.13 Hz range (FB1-FB2), with some variations between directions and age groups (see Tables 2 and 3). The greater impact of VR on older adults was primarily reflected by a significantly higher spectral energy in the 0.4–8.5 Hz frequency range (FB5-FB8) and a concurrent reduction in energy within the 0.03–0.27 Hz frequency range (FB1 and FB3). However, repeated VR sessions led to significant adaptations. Spectral energy in the 0.4–8.5 Hz range (FB5-FB8) decreased in both directions, while energy in the 0.03–0.07 Hz range (FB1) increased in the lateral direction. Interestingly, these adaptations were larger for the older group with a larger decrease in FB8 and a larger increase in FB1 in anteroposterior direction, and larger increase in FB1-FB3 in the lateral direction, compared to the young adults. These results suggest that older people rely more on visual input and therefore react more strongly to the visual perturbation. The more pronounced adaptation among the older group is likely due to the relatively larger initial reaction to VR-stimulation. Taken together, both young and older adults showed similar adaptation to visual stimulation via VR, with a shift from slower to higher frequency bands. However, this shift was more pronounced among people of older age.

The decomposition of the CoP signal into frequency-specific components provides a useful, albeit indirect, method to identify the organization of postural control. Lower-frequency oscillations are commonly interpreted as reflecting slower, more global control processes, potentially involving sensory integration and exploratory sway, whereas higher-frequency components are often associated with faster, feedback-driven corrective actions and increased muscle activation and co-activation to increase joint stiffness (impedance)^8^. Within this framework, the distribution of spectral power observed in the present study suggests that visual perturbations engage multiple control mechanisms operating across different timescales. Subtle differences between groups in the relative contribution of these frequency ranges may reflect differences in control strategy, such as a greater reliance on rapid corrective adjustments versus slower, visually mediated regulation. However, these interpretations should be made with caution, as frequency-domain measures of CoP provide only indirect evidence of underlying neural and biomechanical processes. Spectral analysis with complementary measures (such as muscle activity or kinematics)^31^ would have been valuable here to more directly link frequency-specific sway behavior to mechanisms of postural control.

Collectively, our findings suggest that the postural control system adjusts its behavior in response to changing visual input, likely reflecting adaptive processing of visual information during stance. The findings also highlight the potential of VR-based rehabilitation involving artificial balance perturbations as a promising approach for enhancing resilience to visually induced instability. These findings may have implications for populations such as older adults, who are often reported to rely more heavily on visual inputs. However, as the present study did not include clinical or impaired groups, this extrapolation of findings to clinical populations remains speculative and should be confirmed in future research. Older individuals can experience substantial postural instability when sensory information is erroneous or conflicting, and this may lead to a fall. However, postural stability could be improved by isolating the sensory dysfunction and recalibrating the CNS with targeted rehabilitation of specific sensory inputs. This study demonstrated that, when exposed to a perturbing, immersive, visual environment, repeated exposures effectively promote sensory recalibration and a change in postural strategy in both healthy young and older adults. These adaptations facilitated better utilization of more reliable sensory resources, contributing to improvements in postural control and increased resilience to balance perturbations induced by visual motion. However, a limitation of this study is that there is no long-term evaluation of the immersive environment. Thus, five trials of the immersive environment may not be sufficient to promote long-standing changes to the postural control system.

This study incorporated two control conditions, quiet stance with eyes closed and quiet stance with eyes open to examine the role of vision in a “passive mode”, i.e., when visual input was either absent or congruent with other sensorimotor sources rather than in conflict with them. Notably, the differences observed between the EC and EO conditions, in terms of the distribution of the most affected frequency bandwidths by the test condition, closely mirrored those observed when comparing VR exposure to quiet stance with eyes open. This suggests that the destabilizing effects of immersive VR may mimic the removal of visual input in terms of spectral changes in some respects, thereby reinforcing the idea that VR environments can serve as effective tools for probing the role of visual information in postural control. That said, the relative sizes of the spectral power used within these key frequency bandwidths during tests with EC and during VR stimulation were not compatible. Our findings suggest that visual motion may pose a larger challenge to postural control than having no visual information at all with EC. While some features of postural control under VR exposure resembled those observed in conditions with EC, it is important to note that dynamic visual stimulation (e.g., optic flow and vection) represents a fundamentally different sensory context than the absence of vision. Rather than mimicking the EC condition, VR-based perturbations likely introduce conflicting or misleading visual cues.

Although the group of older adults was presented with a less challenging VR stimulation, we could see larger shifts in the spectral power distribution due to VR compared to the young group. This is important knowledge for development of balance interventions. The VR-allows for a graded visual stimulation, from small to large perturbations. How to optimally present visual disturbances via VR in balance training to the individual’s function and daily activities need to be evaluated in future research. Age-related differences in postural responses to visual perturbation observed in the present study are consistent with previous research demonstrating altered sensory integration in older adults. Experimental work using moving visual scenes and sensory organization paradigms has shown that older adults tend to rely more heavily on visual information and exhibit reduced flexibility in sensory reweighting under conditions of sensory conflict^4,32–34^. Furthermore, age-related increases in reliance on ankle stiffness and changes in neuromuscular control strategies have been reported, particularly under conditions of uncertainty^35–37^.

Several components of the sensorimotor system are likely compromised with aging, including reduced capacity for rapid muscle force generation and elevated thresholds for detecting sensory input^15^. Additionally, the results suggest that performance during quiet stance control tests may provide some insight into how individuals respond to more complex or dynamic balance perturbations. Notably, the use of wavelet analysis in this study offered a more nuanced understanding of postural control dynamics, revealing spectral features that are often overlooked by conventional posturography analysis methods.

One limitation of this study is that it focused solely on the short-term effects of VR exposure, specifically, the outcomes achievable through repeated training over the course of a single hour within one day. Our findings suggest that performing this kind of repeated training was able to reduce the relative power used within the most affected spectral bandwidths by about 40% (young group) respectively 50% (older group). However, we also acknowledge that when the values do not return to baseline levels this may indicate a sustained effect within the timeframe assessed. Accordingly, five repeated VR training sessions were not sufficient to make either the young or older group fully resilient to immersive visual environments. However, for rehabilitation strategies to be effective in real-world settings, it is essential that improvements in sensorimotor control strategies are consolidated over time and transferred to daily activities, allowing the benefits to be retained during future exposures to similar postural challenges^21,22^. Addressing this important aspect of long-term retention and transfer of learning should be a key objective in future VR-based postural control studies.

Another limitation is that, although participants significantly reduce the destabilizing effects of the VR environment within five sessions, few of the posturography parameters, particularly among older adults, suggested that performance had returned to baseline levels observed during control tests with eyes open. This suggests that while short-term adaptation is possible, there remains substantial potential for further improvement. Achieving this may require more advanced or prolonged training protocols, such as repeated sessions conducted multiple times per week and the use of varied VR content to promote broader generalization of sensorimotor adaptations.

Moreover, it should be noted that different VR stimuli were presented to the younger and older groups to mitigate discomfort in older participants. Whilst this approach was taken for ethical and practical reasons, it introduces a potential confound, as differences in stimulus intensity or visual motion characteristics may have influenced the observed group differences. As such, comparisons between groups should be interpreted with caution, as they may reflect both age-related factors and differences in stimulus exposure. That said, the most likely consequence of this limitation is that the older group responded less to the VR stimulation than they would have if they had viewed the same VR movie as the younger group. Therefore, the reported results likely underestimate the true effects of VR on older adults. However, although this study limitation likely raised the threshold for detecting age-related differences, the test conditions were sufficiently appropriate to reveal numerous significant age-related differences for adaptation and power spectra patterns. For example, older adults group displayed more accentuated responses than the young group, specifically in the lateral direction, suggesting that the ability to handle distorted visual information might be affected by age-related factors. This study was not designed to address the underlying reasons why older adults appear more prone to multidimensional instability. However, our findings are consistent with previous literature indicating that, compared to younger adults, older individuals more frequently respond to balance perturbations with increased instability in both the anteroposterior and mediolateral directions, even when the perturbations are primarily unidirectional^25,38–40^.

A wavelet analysis can be performed according to different model approaches (e.g., ’Continuous Wavelet Transforms’ or ’Maximal overlap discrete wavelet transforms’)^41,42^. The number of wavelets extracted and their spectral characteristics (e.g., based on Coiflet settings) can also be adjusted. We found that our wavelet analysis configuration was successful in systematically revealing several important features in human postural control from force platform posturography recordings, e.g., adaptation-related changes and response pattern differences between healthy young and older adults. It was beyond the scope of this study to explore the effects of using alternative wavelet analysis configurations, but the role of analysis model design might be an aspect to explore in future research.

Finally, it should be noted that the participants in this study were either young adults or relatively healthy older adults. In contrast, the primary clinical target group for VR-based rehabilitation includes patients with central nervous system disorders or other sensorimotor impairments. In such individuals, one or more alternative sensory systems to vision, such as tactile, proprioceptive, or vestibular systems, may already be compromised due to pathological or age-related degeneration. This could render patients more susceptible to destabilization when exposed to immersive visual environments. The findings of this study underscore the importance that when the challenges imposed by VR-based stimuli used for rehabilitation remain within tolerable limits for each individual patient even people of older age can adapt. Furthermore, a key objective for future research will be to develop and evaluate customized rehabilitation protocols tailored to the specific needs and capacities of different patient populations.

Wavelet analysis indicates that younger and older adults adopted similar spectral response patterns when responding to immersive visual stimulation. However, older adults exhibited larger shifts in spectral characteristics, indicating age-related differences in resilience to visual disturbances. The initial exposure to the VR environment produced a comparable, pronounced dual-component spectral response in both groups, where spectral energy increased significantly in the high-frequency range (0.4–8.5 Hz), while a significant decrease occurred in the anteroposterior direction within the low-frequency range (0.03–0.27 Hz). Notably, these responses were not concentrated in the low-frequency band typically associated with visual input, indicating that postural control does not function as a simple linear input–output system in the spectral domain. In older adults, the added challenge of VR was primarily reflected in elevated spectral energy within the high-frequency range. Overall, the findings suggest that postural control relies on dynamic multisensory integration, continuously reweighting sensory inputs based on their reliability rather than adhering to a fixed spectral response pattern. Accordingly, VR training may enhance resilience to immersive visual environments by reducing the impact of disruptive sensory information.

## Materials and methods

### Ethical consideration

The study was performed in accordance with the latest Declaration of Helsinki and received ethical approval from the Ethical Review Authority in Sweden (Dnr:2023-00353-01). All participants gave their written signed consent prior to participation.

### Participants

The participants consisted of two groups. The group denoted as “Young” consisted of 28 participants within the range of 20–41 years (17 males; mean age 25.3 years, (standard deviation (SD)) 4.6 years). The group denoted as “Older” consisted of 25 participants older than 65 years (14 males; mean age 74.8 years, SD 3.5 years). The participants were recruited using approved social media advertisements, and through connections with friends and family. Participants were healthy and had no prior history of diseases that could affect their balance, e.g., a known history of vertigo or neurological diseases. A custom questionnaire was used to attain relevant medical history and screen any existing morbidities that could preclude inclusion into the study. The investigations were performed at the Movement and Reality Laboratory (MoRe-lab), Lund University, Sweden.

### Test procedure

All investigations were carried out on the same day and required approximately 90 min per person. The VR headset used was an Oculus Quest 2 (Meta Technologies, LLC, Menlo Park, California, USA). A Quest 2 VR headset weighs about 503 g, which corresponds to the weight of a standard construction helmet. This VR device tracks the head movements in 360-degree range and makes any recorded change in head direction cause an identical change of direction in the VR movie displayed to the subject. First, the participants viewed a 360° video while sitting to calibrate the sharpness of the images displayed through the VR visor. Thereafter, the participants were instructed to stand on a force platform without shoes (socks were permitted), with their feet positioned with a slight outward rotation (approximately 30 degrees) using guidelines on the force platform. This was done to standardize the posturography test protocol^18,25,43^. The participants were thereafter instructed to stand in a relaxed position, with their arms folded over their chests and to look straight forward without moving the head during all tests. An AMTI force platform (AMTI HPS 464508, AMTI Europe GmbH, Helmstadt-Bargen, Germany) was used for recording the participants’ postural stability with a sample rate of 1000 Hz.

The participants performed seven posturography tests. Two control tests (quiet stance with eyes open, quiet stance with eyes closed), were performed in a randomized order before the five VR sessions were performed. During the control test eyes open, the participants were instructed to focus on a visual target positioned at eye level on the wall 5 m in front of them. The control tests were used for assessing the normal postural sway and to provide a relative reference compared to tests with distortive visual information from VR. Participants did not wear the VR headset when performing the control tests, since the VR equipment precluded proper visual feedback.

Five assessments were made while exposed to the VR stimuli, repeatedly watching a 360° video of a roller-coast ride, where the ride included numerous quick turns to the right and left and ascending/descending elevations. A VR movie must possess specific immersive qualities to evoke an internal sense of motion while maintaining the perception that the movement is not self-generated. After evaluating numerous VR films, we found that only a small set of real-life recordings met these criteria, and among them, rollercoaster scenes were particularly suitable, as they depict a situation most people have experienced and could evoke an internal sense of motion. The VR stimuli used in this study were similar in nature to those we have previously employed to perturb balance^18^. The same 120-second segment from the video was shown in every session to the same individual. The young and older adult groups watched similar but not identical VR movies to somewhat mitigate the visual strain that the older adult group were exposed to. The video that the older adult group watched included slower speed and fewer velocity changes. The participants were allowed to sit down and rest for 10 min between repeated VR sessions. Illustrations of postural control responses evoked by watching an immersive VR movie can be found in the paper (Fransson et al. 2019)^18^.

### Posturography analysis

The stability measures and adaptations in anteroposterior and lateral directions during repeated VR sessions and during the control tests were determined by analyzing the center of pressure (CoP) recordings using the wavelet method - maximal overlap discrete wavelet transform - with Coiflet set to 1 and the number of wavelets to be obtained set to 16 (i.e., determining the wavelets bandwidths analyzed). The wavelet analysis was performed with the MATLAB 2023a software (The Mathworks Inc, Natick, Massachusetts, USA) using the function “MODWT” with the wavelet Coiflet. The sum of all wavelets was thereafter normalized to be 100 (i.e., the values presented in the figures are without sort). Finally, the power of each wavelet was determined by multiplying the normalized wavelet value with the spectral size encompassed by the wavelet. The “MODWT” MATLAB function automatically generates wavelets for 16 different frequency bands based on the selected input parameters defining the algorithm’s settings. Of these, 8 wavelets were included in the final analysis. The lower limit for the frequency range was defined by the 120-second trial duration, ensuring that at least three full oscillations were represented within the wavelet decomposition. The upper limit was determined using FFT results, which indicated that activity above a certain frequency mainly reflected “white noise”. The frequency bandwidths of the 8 wavelets analyzed were: FB1 = 0.028–0.067 Hz; FB2 = 0.055–0.133 Hz; FB3 = 0.110–0.266 Hz; FB4 = 0.219–0.531; FB5 = 0.438–1.060 Hz; FB6 = 0.88–2.12 Hz; FB7 = 1.75–4.25 Hz; and FB8 = 3.51–8.50 Hz.

### Statistical analysis

The stability in anteroposterior and lateral directions, as reflected by log-transformed wavelet values, were analyzed using repeated measures GLM ANOVA. The analysis method was used after ensuring that all dataset combinations analyzed in the study with this statistical method produced model residuals that had normal or close to normal distribution, thus validating the appropriateness of GLM ANOVA for analyzing the datasets in this study^44,45^.

The main factors and factor interaction analyzed for VR sessions were: ‘Repetition’ (Session 1…5; degree of freedom (d.f.) 4)), ‘Age group’ (Young vs. Older; (d.f.) 1)), and the main factor interaction ‘Repetition’ x ‘Age group’. In the analyses, the log-transformed wavelet values were dependent variables, the model parameter ‘Repetition’ was a within-subjects independent variable and the ‘Age group’ was a between-groups independent variable.

The main factors and factor interaction analyzed for the control tests were: ‘Vision’ (Eyes closed vs. Eyes open; d.f. 1), Age group’ (Young vs. Older; (d.f.) 1)), and the main factor interaction ‘Vision’ x ‘Age group’. In the analyses, the log-transformed wavelet values were dependent variables, the model parameter ‘Vision’ was a within-subjects independent variable and the ‘Age group’ was a between-groups independent variable.

The Wilcoxon matched-pairs signed-rank test (Exact sig. 2-tailed)^46^ was used for post hoc within-subjects analyses for each age group to further characterize the effects of the main factors identified in the repeated-measures GLM ANOVA. Comparisons were made between VR Session 1 and VR Session 5 to assess the effects of repeated VR exposure; between VR Session 1 and the EO control condition to evaluate the initial effect of VR; and between VR Session 5 and the EO control condition to assess the effects following repeated exposure. Post-hoc within-subject comparisons were also conducted to examine the influence of visual input in the control test^26^.

In the GLM ANOVA analyses, p-values *p* < 0.05 were considered significant. In the post-hoc analyses of VR session data, p-values < 0.025 were considered significant after Bonferroni correction^46^. In the post-hoc analyses of control test data, p-values < 0.05 were considered significant after Bonferroni correction. Non-parametric statistical methods were used in the post hoc tests as the Shapiro-Wilk test revealed that some datasets were not normally distributed, and that normal distribution could not be obtained by log-transformation^46^. The statistical analyses were performed using SPSS (Version 28, IBM Corp, Armonk, New York, USA).

## Data Availability

Data is available upon request to the authors.
